# An RT-qPCR Assay from Rectal Swabs for the Detection of Rabbit Hemorrhagic Disease Virus 2 in Natural Cases

**DOI:** 10.1155/2023/1869692

**Published:** 2023-03-24

**Authors:** Javier Asin, Megan E. Moriarty, Andrea B. Mikolon, Deana L. Clifford, Daniel Rejmanek, Francisco A. Uzal, Beate M. Crossley

**Affiliations:** ^1^California Animal Health and Food Safety Laboratory, University of California-Davis, Davis (DR, BMC) and San Bernardino (JA, FAU), CA, USA; ^2^Wildlife Health Laboratory, California Department of Fish and Wildlife, Rancho Cordova, CA, USA; ^3^Department of Medicine and Epidemiology and Karen C. Drayer Wildlife Health Center, School of Veterinary Medicine, University of California-Davis, Davis, CA, USA; ^4^California Department of Food and Agriculture, Sacramento, CA, USA

## Abstract

Rabbit hemorrhagic disease virus 2 (RHDV2 or *Lagovirus europaeus* GI.2) is spreading across North America. This has enabled submissions of lagomorphs for testing to veterinary diagnostic laboratories (VDLs). The liver is currently the gold-standard sample type for testing by RT-qPCR. VDL clients usually seek alternate diagnostic approaches that permit simpler and faster sample collection with less risk of environmental contamination; there is also a necessity for a sample type that can be collected from live animals. Therefore, the goal of this study was to optimize and evaluate an RT-qPCR assay on rectal swabs collected from a group of carcasses of leporids of different species that were submitted to a VDL during an RHDV2 outbreak. A total of 130 carcasses were tested both by liver tissue and rectal swab RT-qPCR. The results of the liver samples were considered the gold standard, and 73 carcasses tested positive and 57 carcasses tested negative in liver. Out of the 73 liver RT-qPCR-positive carcasses, 64 tested positive and 9 tested negative on the rectal RT-qPCR. All 57 liver RT-qPCR-negative carcasses tested negative on the rectal RT-qPCR. The sensitivity and specificity of the rectal RT-qPCR were 88% and 100%, respectively, most likely due to significantly (*p* < 0.001) lower viral loads in the rectal swabs (median Ct: 27.03) compared to the liver samples (median Ct: 12.69). Despite being more than 4 logs less sensitive, RT-qPCRs from rectal swabs can be used to screen leporid carcasses for the presence of RHDV2 RNA.

## 1. Introduction

Rabbit hemorrhagic disease virus 2 (RHDV2 or *Lagovirus europaeus* GI.2) is a single-stranded RNA virus within the genus *Lagovirus*, family *Caliciviridae*, that causes a high-mortality illness characterized by hepatic necrosis in several rabbit (*Oryctolagus cuniculus*, *Sylvilagus* spp) and hare (*Lepus* spp) species [1]. As the virus spreads to some previously free areas such as North America, new lagomorph species are being exposed, and thus, the known viral host range is widening [2, 3]. The latter entails conservation concerns, since some potentially susceptible autochthonous lagomorph species may be endangered. In addition, such a wide host range facilitates spillover events between wild and domestic lagomorphs, and vice versa [4], which complicates disease control and makes the occurrence of seasonal outbreaks almost inevitable.

Veterinary diagnostic laboratories (VDLs) play an important role in the management of outbreaks by providing high-quality and rapid RHDV2 diagnosis, most often using the gold standard test, liver RT-PCR or RT-qPCR [1]. However, this test usually involves shipping whole carcasses to a local or distant VDL or performing necropsies for liver collection at the field or in the affected premises, which increases the risk of environmental contamination and disease spread. Rabbit owners, veterinary practitioners, wildlife and agricultural management agencies, and other VDLs clients are constantly seeking alternate, less invasive diagnostic approaches that can also be used as screening methods [5]. Various studies have shown that, in addition to liver, RHDV2 RNA can be detected by RT-qPCR in blood, urine, and oral or rectal swabs in experimental cases [6–9]. In particular, rectal swabs are a valuable and easily obtainable sample type because postmortem sample collection does not involve opening the carcass, and antemortem sample collection is minimally invasive. For these reasons, we hypothesized that this could be a suitable diagnostic sample for viral detection in natural cases of the disease in both domestic and wild leporids. Therefore, the aim of this study was to develop and evaluate an RT-qPCR assay on rectal swabs taken from carcasses of different species of leporids that died during an outbreak of RHDV2.

## 2. Materials and Methods

### 2.1. Case Selection

A total of 130 carcasses (carcass 1–130) of nonvaccinated domestic and wild leporids that were submitted for diagnostic purposes to the California Animal Health and Food Safety Laboratory System (CAHFS; University of California-Davis) during the 2020-2021 RHDV2 outbreak in California, USA, were selected. For sample size calculation, the guidelines of the American Association of Veterinary Laboratory Diagnosticians for a method comparability study (i.e., liver RT-qPCR versus rectal swab RT-qPCR) were followed [10].

### 2.2. Liver and Rectal RT-qPCR

For liver tissue samples and rectal swabs obtained during necropsies, RT-qPCR using a one-step assay kit (TaqMan Fast Virus 1-Step; Thermo Fisher) targeting the *vp60* gene of RHDV2 was used as previously described [11, 12]. The rectal swabs were placed in 3 mL of Dulbecco's modified eagle's medium and kept refrigerated until processed. A total of 200 *μ*L of rectal swab material were subjected to RNA extraction following the manufacturer's instructions and as described for the liver [12]. For both sample types, cycle threshold (Ct) values below 35 were considered positive, and Ct values over 35 were considered negative. Normality of the distributions of the liver and rectal swab positive Ct values were analyzed by means of the Kolmogorov–Smirnov test, and the medians were compared via the Wilcoxon test for paired samples. *p*-values <0.05 were considered significant. Statistical analysis was performed using IBM SPSS 29.0 (IBM Corp., Armonk, NY).

### 2.3. Calculation of Sensitivity and Specificity of Rectal RT-qPCR

The liver RT-qPCR status (positive or negative) was used as the gold standard for the calculation of sensitivity and specificity of the rectal RT-qPCR. Sensitivity was defined as the percentage of liver RT-qPCR-positive carcasses that were positive on rectal swabs. Specificity was defined as the percentage of liver RT-qPCR-negative carcasses that were negative on rectal swabs.

### 2.4. Calculation of Positive and Negative Predictive Values of the Rectal RT-qPCR

Positive predictive value was defined as the proportion of rectal RT-qPCR-positive carcasses that were correctly identified as positive on liver RT-qPCR. Negative predictive value was defined as the proportion of rectal RT-qPCR negative carcasses that were correctly identified as negative on liver RT-qPCR.

### 2.5. Comparison of Liver and Rectal Swabs RT-qPCR Ct Values among Species and Age Groups

In the liver RT-qPCR-positive carcasses, liver and rectal swabs Ct values were compared between the different species that tested positive pooled in three groups by genera (i. *Oryctolagus* sp. [domestic rabbit]; ii. *Lepus* sp. [black-tailed jackrabbit]; iii. *Sylvilagus* spp. [desert cottontail and brush rabbit]). When available, the age of the liver RT-qPCR-positive domestic rabbits was recorded, and three groups were established, as reported recently by Tu et al. [9]: kits (4 weeks or less), young or subadults (5 to 24 weeks), and adults (25 weeks or more). The liver and rectal swabs Ct values were compared among the three age groups; when the age was not available, the case was excluded from the analysis.

For the different groups in both data subsets (genera and age), normality and equality of variance were tested by means of the Shapiro–Wilk and Levene's tests, respectively. Paired comparisons between the different groups were performed via the Mann–Whitney *U* test (if the data did not follow a normal distribution) or Student's *t*-test (if the data followed a normal distribution [alternatively, Welch's *t*-test was used if the variances were not equal]). *p*-values <0.05 were considered statistically significant. Statistical analyses were performed using IBM SPSS 29.0.

### 2.6. Additional Tests Performed in Selected Cases

For all the liver RT-qPCR-positive carcasses, hematoxylin and eosin-stained histological sections of the liver were prepared routinely and examined using light microscopy to determine the presence/absence of necrosis compatible with lagovirus infection [13]. Sections of selected liver RT-qPCR positive carcasses were stained with pan-lagovirus immunohistochemistry (IHC; 6G2, 3H6, and 6D6 mouse monoclonal antibody cocktail; RHD OIE reference laboratory, Brescia, Italy), as previously described [12, 14].

## 3. Results and Discussion

Results of the liver and rectal RT-qPCR are shown in Table 1 and in Supplementary Tables S1 and S2. The sensitivity and specificity of the rectal RT-qPCR were 88% and 100%, respectively. The median Ct values of the positive samples were significantly (*p* < 0.001) lower in the liver (median: 12.69; interquartile range [IQR]: 11.52–14.02; minimum: 9.22; maximum: 31.71) than in the rectal swab (median: 27.03; IQR: 25.28–29.53; minimum: 18.11; maximum: 34.38). These values are consistent with a much lower viral load (more than 4 logs) in the rectal samples and concur with the results obtained in a recent experimental infection in New Zealand white rabbits (*Oryctolagus cuniculus*) and Eastern cottontails (*Sylvilagus floridanus*) [8], in which RHDV2 RNA was detected in rectal swabs but with a lower viral load than in the liver. The liver is the target organ of pathogenic lagoviruses and the tissue with the highest viral load detected consistently in most studies including natural or experimental cases [1]. The positive predictive value for the rectal RT-qPCR was 100%, and thus, a positive rectal swab RT-qPCR was a perfect predictor of a positive liver RT-qPCR result. However, the negative predictive value for the rectal RT-qPCR was 86%, indicating that 14% of the times rectal swab RT-qPCR results were false negatives. Predictive value is dependent on the true disease prevalence (estimated in this study using the gold-standard liver RT-qPCR), which was 56% in this sample population of wild and domestic leporids.

RHDV2 RNA has been detected in fecal pellets of infected rabbits before, both in experimental settings and in cases of natural disease [9, 15, 16]. It is likely that some of the positive results obtained on the rectal swabs were due to viral RNA present in fecal material from the rectum or around the anus. As such, it would be advisable to ensure that some fecal material impregnates the tip of the swab when this sample is collected in order to foster viral detection and avoid false negatives. The contribution of other secretions and tissues, such as blood [8, 9], mucus, and/or sloughed rectal mucosa to the detections, is also very likely. In fact, RHDV2 RNA has been detected in a variety of organs, including several intestinal segments [14], and we have detected it previously during the California RHDV2 outbreak in other tissues of naturally infected animals, such as skeletal muscle (data not shown).

The 73 liver RT-qPCR positive carcasses (carcass 1–73; Suppl.  Table 1) included 55 domestic rabbits (*Oryctolagus cuniculus*), 12 desert cottontails (*Sylvilagus audubonii*), 5 black-tailed jackrabbits (*Lepus californicus*), and 1 brush rabbit (*Sylvilagus bachmani*). The 57 liver RT-qPCR negative carcasses (carcass 74–130; Suppl.  Table 2) included 33 domestic rabbits, 13 desert cottontails, 4 black-tailed jackrabbits, 4 wild rabbits of not specified species (*Sylvilagus* sp.), and 3 brush rabbits (including 1 individual of the subspecies riparian brush rabbit [*S. bachmani riparius*]). The 9 carcasses that tested positive on liver but negative on rectal swab included 8 domestic rabbits (carcasses 3, 18, 42, 46, 54, 57, 64, 65; Suppl.  Table 1) and 1 desert cottontail (carcass 25; Suppl.  Table 1). Therefore, the rectal swab assay demonstrated a good performance not only in domestic rabbits but also in wild leporids native to the American continent, since it was able to detect positive individuals of the three different species (desert cottontail, brush rabbit, and black-tailed jackrabbit) included in the study. Despite the lower overall sensitivity and the fact that only dead individuals were used in this work, our results suggest that the rectal swab assay could be used to screen and monitor for the presence of RHDV2 in live animals, such as wild lagomorphs captured as a part of vaccination strategies in specific geographical areas [17]. Rectal swabs can also be used routinely as part of such vaccination strategies to assess viral shedding in vaccinated individuals, with the caveat that viral load may be lower in comparison to nonvaccinated, infected leporids [7]. In certain circumstances, rectal RT-qPCR might be considered as a useful test prior to the international or interstate movement of rabbit carcasses. In addition, this assay could be used as an *in vivo* test in pet rabbits with chronic presentations [18]. This, however, needs to be tested appropriately with live individuals, since some experimental settings have shown that rectal shedding starts decreasing as early as 2 to 7 days after the challenge [6].

The positive liver RT-qPCR Ct values were significantly higher in the *Oryctolagus* sp. group (median Ct value: 13.02; IQR: 12.27–14.11) than in the *Lepus* sp. (median Ct value: 11.51; IQR: 10.59–11.88; *p* = 0.007) and *Sylvilagus* spp. (median Ct value: 11.27; IQR: 10.70–12.01; *p* < 0.001) groups. The viral loads in the liver of the last two groups were thus higher than in the domestic rabbits; however, the number of liver-positive* Oryctolagus* sp. carcasses (*n* = 55) was considerably higher than those of positive *Lepus* sp. (*n* = 5) and *Sylvilagus* spp. (*n* = 13) carcasses. Additional studies that compare the liver Ct values with a similar number of animals per group are warranted in order to determine whether the wild species have consistently higher viral loads. There were no significant differences in the positive rectal RT-qPCR Ct values between the three genera groups.

The age was available in 50 out of 55 liver RT-qPCR-positive domestic rabbit carcasses. Of those, 23 were adults, 23 were subadults, and 4 were kits; in addition, 22 adults, 18 subadults, and 2 kits were rectal RT-qPCR positive. There were no significant differences in the liver or rectal RT-qPCR Ct values between the three age groups. This differs from the results of a recent experimental study that reported significantly higher viral loads in the liver of 4-week-old kits compared with 13-week-old (subadult) and 25-week-old (adult) rabbits [9]. The low number of kits in our study population could have prevented us from observing a statistical difference. In addition, our study subjects were natural cases of the disease for which the duration and progression of the infection were unknown.

Out of the total 73 liver RT-qPCR positive carcasses, 69 had hepatic necrosis typical of lagovirus infection histologically; in 2 out 73, the presence or absence of hepatic necrosis could not be unequivocally determined due to autolysis, but the liver sections were positive by IHC; in the remaining 2 out of 73, necrosis was not detected and IHC was negative (Suppl.  Table 1). These two carcasses (18 and 65) had low viral loads with liver Ct values of 31.71 and 28.36, respectively, and negative rectal RT-qPCRs. They were less than 1-week-old (estimated by size) domestic rabbits that originated from outbreaks that occurred in a large rabbit meat farm and in a backyard breeder premises, respectively, and they were submitted together with other positive (both carcasses) and negative (in the case of carcass 18, only) carcasses from those locations. It is possible that these two kits died due to other causes, such as starvation or hypothermia (i.e., due to death of the doe during the outbreak), before active viral replication could be established, and thus low viral loads and no lesions or antigen were detected in the liver. Interestingly, five other carcasses (98–102; Suppl.  Table 2) of very young kits, which were collected at the same time as carcass 18 during the outbreak in the large meat farm, and that either died or were culled by the farmer, tested negative, which would support the previous hypothesis. In order to rule out cross-contamination of these two samples in the laboratory, the RNA extractions and liver RT-qPCRs were repeated from the same samples with similar results. Cross-contamination on the necropsy floor was unlikely since basic measures (i.e., working under a biosafety hood or in an isolation room; frequent cleaning and disinfection of surfaces; change of gloves, tools, and surface between necropsies in multicarcass submissions) were always maintained, but cannot be definitively excluded.

One potential limitation of this study is the fact that leporids of different species were grouped together, and wild species (desert cottontails, black-tailed jackrabbits, brush rabbits, and wild rabbit species not specified) were moderately underrepresented compared to domestic rabbits. However, these proportions were representative of the caseload in our VDL system during the RHDV2 outbreak, and no more individuals of the wild species were available for testing. Nevertheless, it is possible that some aspects of this disease could vary in the different species, especially in leporids native to the American continent for which very scant data are currently available. Additional similar studies with larger sample sizes from single leporid species could refine this aspect and determine if the obtained sensitivity and specificity of the rectal RT-qPCR in this group of species are representative of each one separately. In this line, it is possible that true disease prevalence in wild species may be lower than the prevalence observed in this study population, therefore resulting in a lower positive predictive value and a higher negative predictive value; it is also plausible that disease prevalence may be higher among domestic rabbits, particularly those in close contact (i.e., in rabbitries), in which case the positive predictive value would be higher and the negative predictive value would be lower than that of this sample population. Additional field and laboratory studies are needed to further evaluate this diagnostic tool for different species, demographic groups, and clinical contexts.

## 4. Conclusion

RT-qPCR from rectal swabs can be used as a rapid method to screen leporid carcasses of different species for the presence of RHDV2 RNA with high specificity and slightly lower sensitivity than RT-qPCR from liver samples.

## Figures and Tables

**Table 1:** Number of positive and negative individuals on liver and rectal RT-qPCR.

	Liver pos	Liver neg	Total
Rectal swab pos	64	0	64
Rectal swab neg	9	57	66
Total	73	57	130

pos: positive; neg: negative.

## Data Availability

The data supporting the current study are available as supplementary materials and from the corresponding author upon request.

## Supplementary Materials

Supplementary Materials Supplementary Table 1. Results in positive (i.e., RT-qPCR liver positive) cases (carcasses 1–73). Supplementary Table 2. Results in negative (i.e., RT-qPCR liver negative) cases (carcasses 74–130).

## References

[1] Capucci L., Cavadini P., Lavazza A., Bamford D. H., Zuckerma M. (2021). Rabbit hemorrhagic disease virus and European brown hare syndrome virus. *Encyclopedia of Virology*.

[2] Lankton J. S., Knowles S., Keller S., Shearn-Bochsler V. I., Ip H. S. (2021). Pathology of lagovirus europaeus GI.2/RHDV2/b (rabbit hemorrhagic disease virus 2) in native North American lagomorphs. *Journal of Wildlife Diseases*.

[3] Asin J., Nyaoke A. C., Moore J. D. (2021). Outbreak of rabbit hemorrhagic disease virus 2 in the southwestern United States: first detections in southern California. *Journal of Veterinary Diagnostic Investigation*.

[4] Le Gall-Reculé G., Lemaitre E., Bertagnoli S. (2017). Large-scale lagovirus disease outbreaks in European brown hares (Lepus europaeus) in France caused by RHDV2 strains spatially shared with rabbits (Oryctolagus cuniculus). *Veterinary Research*.

[5] Jennings-Gaines J. E., Luukkonen K. L., Robbins K. M. (2022). Utilizing blood filter paper and ear punch samples for the detection of rabbit hemorrhagic disease virus 2 by RT-rtPCR. *Journal of Veterinary Diagnostic Investigation*.

[6] Dalton K. P., Balseiro A., Juste R. A. (2018). Clinical course and pathogenicity of variant rabbit haemorrhagic disease virus in experimentally infected adult and kit rabbits: significance towards control and spread. *Veterinary Microbiology*.

[7] Le Minor O., Boucher S., Joudou L. (2019). Rabbit haemorrhagic disease: experimental study of a recent highly pathogenic GI.2/RHDV2/b strain and evaluation of vaccine efficacy. *World Rabbit Science*.

[8] Mohamed F., Gidlewski T., Berninger M. L. (2022). Comparative susceptibility of eastern cottontails and New Zealand white rabbits to classical rabbit haemorrhagic disease virus (RHDV) and RHDV2. *Transbound Emerg Dis*.

[9] Tu T., Zhou Y., Jiang D. (2022). The pathogenicity comparison of Lagovirus europaeus GI.1 and GI.2 strains in China by using relative quantitative assay. *Scientific Reports*.

[10] Toohey-Kurth K., Reising M. M., Tallmadge R. L. (2020). Suggested guidelines for validation of real-time PCR assays in veterinary diagnostic laboratories. *Journal of Veterinary Diagnostic Investigation*.

[11] Duarte M. D., Carvalho C. L., Barros S. C. (2015). A real time Taqman RT-PCR for the detection of rabbit hemorrhagic disease virus 2 (RHDV2). *Journal of Virological Methods*.

[12] Asin J., Rejmanek D., Clifford D. L. (2022). Early circulation of rabbit haemorrhagic disease virus type 2 in domestic and wild lagomorphs in southern California, USA (2020-2021). *Transbound Emerg Dis*.

[13] Marcato P. S., Benazzi C., Vecchi G. (1991). Clinical and pathological features of viral haemorrhagic disease of rabbits and the European brown hare syndrome. *Rev Scie Tech*.

[14] Neimanis A., Larsson Pettersson U., Huang N., Gavier-Widén D., Strive T. (2018). Elucidation of the pathology and tissue distribution of Lagovirus europaeus GI.2/RHDV2 (rabbit haemorrhagic disease virus 2) in young and adult rabbits (Oryctolagus cuniculus). *Veterinary Research*.

[15] Calvete C., Sarto M. P., Iguacel L., Calvo J. H. (2021). Infectivity of rabbit haemorrhagic disease virus excreted in rabbit faecal pellets. *Veterinary Microbiology*.

[16] Calvete C., Sarto M. P., Calvo A. J., Monroy F., Calvo J. H. (2022). Monitoring of rabbit hemorrhagic disease virus in European wild rabbit (Oryctolagus cuniculus) populations by PCR analysis of rabbit fecal pellets. *Journal of Wildlife Diseases*.

[17] Clifford D., Moriarty M., Rudd J. Protecting the endangered riparian brush rabbit from emergent RHDV2 in California, USA.

[18] Abade Dos Santos F. A., Magro C., Carvalho C. L., Ruivo P., Duarte M. D., Peleteiro M. C. (2020). A potential atypical case of rabbit haemorrhagic disease in a dwarf rabbit. *Animals*.

