# Supplementary material for: An RT-qPCR Assay from Rectal Swabs for the Detection of Rabbit Hemorrhagic Disease Virus 2 in Natural Cases
Source: Transbound Emerg Dis. 2023 Mar 24;2023:1869692. doi: 10.1155/2023/1869692 (PMC12017109; doi:10.1155/2023/1869692)
Supplement: Supplementary Materials — Supplementary Table 1. Results in positive (i.e., RT-qPCR liver positive) cases (carcasses 1–73). Supplementary Table 2. Results in negative (i.e., RT-qPCR liver negative) cases (carcasses 74–130). [file 1869692.f1.pdf]

Supplementary materials

Supplementary Table 1. Results in positive (i.e., RT-qPCR liver positive) cases (carcasses 1-73)

Carcass No.	Date tested	Spe-cies	Age Group	Liver PCR	Liver Ct	Rectal PCR	Rectal Ct	Hepatic necrosis	IHC
1	June-2021	DOM	SA	pos	13.02	pos	26.06	yes	ND
2	June-2021	JR	-	pos	11.10	pos	28.02	yes	ND
3	June-2021	DOM	SA	pos	12.89	neg	-	yes	ND
4[a]	June-2021	DOM	SA	pos	13.13	pos	25.66	yes	ND
5[a]	June-2021	DOM	SA	pos	10.92	pos	22.13	yes	ND
6	June-2021	DOM	SA	pos	13.78	pos	28.05	yes	ND
7	June-2021	DOM	SA	pos	12.66	pos	26.05	yes	ND
8	May-2021	DC	-	pos	11.52	pos	21.52	yes	ND
9	May-2021	BR	-	pos	12.03	pos	28.84	yes	ND
10	May-2021	DOM	A	pos	12.88	pos	21.77	yes	ND
11	May-2021	DC	-	pos	9.22	pos	24.48	yes	ND
12	May-2021	DC	-	pos	10.91	pos	23.64	yes	ND
13	May-2021	DC	-	pos	14.09	pos	30.25	yes	ND
14[b]	May-2021	DOM	A	pos	15.16	pos	25.62	yes	ND
15[b]	May-2021	DOM	SA	pos	12.51	pos	28.20	yes	ND
16[b]	May-2021	DOM	SA	pos	12.6	pos	26.20	yes	ND
17[b]	May-2021	DOM	A	pos	13.03	pos	32.28	yes	ND
18[b]	May-2021	DOM	K	pos	31.71	neg	-	no	neg
19[b]	May-2021	DOM	K	pos	13.05	pos	24.53	yes	ND
20[b]	May-2021	DOM	A	pos	14.89	pos	34.38	yes	ND
21	May-2021	DOM	A	pos	11.10	pos	26.40	yes	ND
22	May-2021	DOM	A	pos	10.96	pos	25.07	yes	ND
23	May-2021	DOM	K	pos	13.11	pos	26.00	yes	ND
24	May-2021	DOM	A	pos	9.78	pos	26.74	yes	ND
25	April-2021	DC	-	pos	11.67	neg	-	yes	ND
26	April-2021	DOM	SA	pos	13.17	pos	26.32	yes	ND
27[c]	April-2021	DOM	NA	pos	12.15	pos	27.05	yes	ND
28[c]	April-2021	DOM	NA	pos	14.07	pos	25.05	yes	ND
29[c]	April-2021	DOM	NA	pos	12.61	pos	29.54	yes	ND
30	April-2021	DOM	A	pos	14.22	pos	31.86	yes	ND
31	April-2021	DOM	SA	pos	12.08	pos	25.68	yes	ND
32[d]	April-2021	DOM	A	pos	14.17	pos	19.59	yes	ND
33[d]	April-2021	DOM	A	pos	14.09	pos	30.48	yes	ND
34	April-2021	DOM	A	pos	15.53	pos	27.27	NE	pos
35	April-2021	DOM	SA	pos	13.77	pos	23.33	yes	ND
36	April-2021	DOM	SA	pos	14.68	pos	29.79	yes	ND
37	April-2021	DC	-	pos	12.57	pos	25.72	yes	ND
38	April-2021	DC	-	pos	10.81	pos	18.86	yes	ND
39	April-2021	DOM	NA	pos	13.01	pos	27.20	yes	ND
40	April-2021	DOM	SA	pos	10.56	pos	29.51	yes	ND

41	April-2021	DOM	SA	pos	12.02	pos	29.67	yes	ND
42	April-2021	DOM	A	pos	10.09	neg	-	yes	ND
43[e]	March-2021	DOM	A	pos	14.11	pos	29.18	yes	ND
44[e]	March-2021	DOM	A	pos	13.70	pos	28.51	yes	ND
45	March-2021	DOM	A	pos	12.27	pos	31.08	yes	ND
46	March-2021	DOM	SA	pos	14.25	neg	-	yes	ND
47	March-2021	DOM	A	pos	13.00	pos	28.00	yes	ND
48	March-2021	DOM	A	pos	10.96	pos	18.11	yes	ND
49	March-2021	DOM	A	pos	13.80	pos	26.56	yes	ND
50	March-2021	DC	-	pos	10.59	pos	29.79	NE	pos
51	March-2021	DC	-	pos	11.27	pos	29.02	yes	ND
52	March-2021	DOM	NA	pos	16.22	pos	28.63	yes	ND
53[f]	March-2021	DOM	SA	pos	15.52	pos	25.53	yes	ND
54[f]	March-2021	DOM	SA	pos	21.75	neg	-	yes	ND
55[f]	March-2021	DOM	SA	pos	12.95	pos	28.58	yes	ND
56[f]	March-2021	DOM	SA	pos	13.99	pos	27.00	yes	ND
57[f]	March-2021	DOM	SA	pos	12.90	neg	-	yes	ND
58	March-2021	DOM	SA	pos	11.68	pos	25.20	yes	ND
59[g]	March-2021	DOM	A	pos	12.69	pos	25.53	yes	ND
60	March-2021	DOM	A	pos	14.05	pos	29.77	yes	ND
61	March-2021	DOM	A	pos	12.83	pos	26.95	yes	ND
62	March-2021	DOM	A	pos	12.35	pos	31.37	yes	ND
63	March-2021	DOM	SA	pos	14.32	pos	29.56	yes	ND
64[h]	Feb-2021	DOM	SA	pos	11.05	neg	-	yes	ND
65[h]	Feb-2021	DOM	K	pos	28.36	neg	-	no	neg
66	Feb-2021	JR	-	pos	11.82*	pos	28.56	yes	ND
67	Feb-2021	JR	-	pos	11.51*	pos	33.56	yes	ND
68	Jan-2021	DOM	A	pos	11.80*	pos	24.01	yes	ND
69	Jan-2021	DC	-	pos	11.99*	pos	20.46	yes	ND
70	Dec-2020	JR	-	pos	10.08*	pos	24.93	yes	ND
71	Dec-2020	JR	-	pos	11.93*	pos	29.25	yes	ND
72	Nov-2020	DC	-	pos	11.06*	pos	30.75	yes	ND
73	Nov-2020	DC	-	pos	10.58*	pos	33.17	yes	ND

PCR: RT-qPCR; Ct: Cycle threshold value (positive if ≤35); IHC: immunohistochemistry; DOM: Domestic rabbit (*Oryctolagus cuniculus*); JR: Black-tailed jackrabbit (*Lepus californicus*); DC: Desert cottontail (*Sylvilagus audubonii*); BR: Brush rabbit (*Sylvilagus bachmani*); Age group: age of the domestic rabbits per Tu et al, 2022 (https://doi.org/10.1038/s41598-022-25118-0); A: Adult, ≥ 25 weeks; SA: Subadult, 5 to 24 weeks; K: Kit, ≤4 weeks; pos: positive; neg: negative; ND: not done; NE: not evaluable (i.e., due to autolysis)

[a]Carcasses 4-5 originated from the same premises

[b]Carcasses 14-20 originated from the same premises; also same as carcasses 98-102 (Suppl. Table 2)

[c]Carcasses 27-29 originated from the same premises

[d]Carcasses 32-33 originated from same premises

[e]Carcasses 43-44 originated from same premises; also same as carcass 118 (Suppl. Table 2)

[f]Carcasses 53-57 originated from same premises

[g]Carcass 59 originated from the same premises as carcass 123 (Suppl. Table 2)

[h]Carcasses 64-65 originated from the same premises

*These liver Ct values were reported previously in Asin et al, 2022 (https://doi.org/10.1111/tbed.14315)

Supplementary Table 2. Results in negative (i.e., RT-qPCR liver negative) cases (carcasses 74-130)

Carcass No.	Date tested	Spe-cies	Age Group	Liver PCR	Rectal PCR
74	June-2021	DOM	SA	neg	neg
75	June-2021	JR	-	neg	neg
76	June-2021	DOM	A	neg	neg
77	June-2021	DC	-	neg	neg
78	June-2021	JR	-	neg	neg
79	June-2021	DOM	A	neg	neg
80[i]	June-2021	WRNS	-	neg	neg
81[i]	June-2021	WRNS	-	neg	neg
82	June-2021	DOM	A	neg	neg
83	May-2021	DOM	SA	neg	neg
84[j]	May-2021	DOM	A	neg	neg
85[j]	May-2021	DOM	A	neg	neg
86	May-2021	DOM	SA	neg	neg
87	May-2021	BR	-	neg	neg
88	May-2021	DC	-	neg	neg
89	May-2021	DC	-	neg	neg
90	May-2021	JR	-	neg	neg
91	May-2021	BR	-	neg	neg
92	May-2021	DC	-	neg	neg
93[i]	May-2021	WRNS	-	neg	neg
94[i]	May-2021	WRNS	-	neg	neg
95	May-2021	DOM	A	neg	neg
96	May-2021	DOM	A	neg	neg
97	May-2021	DC	-	neg	neg
98[b]	May-2021	DOM	K	neg	neg
99[b]	May-2021	DOM	K	neg	neg
100[b]	May-2021	DOM	K	neg	neg
101[b]	May-2021	DOM	K	neg	neg
102[b]	May-2021	DOM	K	neg	neg
103	May-2021	DOM	A	neg	neg
104	May-2021	DOM	A	neg	neg
105[k]	May-2021	DOM	K	neg	neg
106[k]	May-2021	DOM	K	neg	neg
107	May-2021	DOM	A	neg	neg
108	April-2021	DOM	A	neg	neg
109[l]	April-2021	DOM	A	neg	neg
110[l]	April-2021	DOM	A	neg	neg
111	April-2021	DOM	NA	neg	neg
112	April-2021	DOM	A	neg	neg

113	April-2021	DC	-	neg	neg
114	April-2021	DC	-	neg	neg
115	April-2021	DC	-	neg	neg
116	April-2021	DOM	SA	neg	neg
117	March-2021	DOM	A	neg	neg
118[e]	March-2021	DOM	A	neg	neg
119	March-2021	DOM	SA	neg	neg
120	March-2021	JR	-	neg	neg
121	March-2021	DC	-	neg	neg
122	March-2021	DC	-	neg	neg
123[g]	March-2021	DOM	A	neg	neg
124	March-2021	DOM	A	neg	neg
125	March-2021	DC	-	neg	neg
126	Feb-2021	DOM	A	neg	neg
127	Feb-2021	DOM	A	neg	neg
128	Feb-2021	DC	-	neg	neg
129	Dec-2020	RBR	-	neg	neg
130	Dec-2020	DC	-	neg	neg

PCR: RT-qPCR; DOM: Domestic rabbit (*Oryctolagus cuniculus*); JR: Black-tailed jackrabbit (*Lepus californicus*); DC: Desert cottontail (*Sylvilagus audubonii*); BR: Brush rabbit (*Sylvilagus bachmani*); RBR: Riparian brush rabbit (*Sylvilagus bachmani riparius*); WRNS: Wild rabbit not specified species (*Sylvilagus* sp.); Age group: age of the domestic rabbits per Tu et al, 2022 (https://doi.org/10.1038/s41598-022-25118-0); A: Adult, ≥ 25 weeks; SA: Subadult, 5 to 24 weeks; K: Kit, ≤4 weeks; neg: negative

[b]Carcasses 98-102 originated from the same premises; also same as carcasses 14-20 (Suppl. Table 1)

[e]Carcass 118 originated from the same premises as carcasses 43-44 (Suppl. Table 1)

[g]Carcass 123 originated from the same premises as carcass 59 (Suppl. Table 1)

[i]Carcasses 80-81, 93-94 originated from the same premises

[j]Carcasses 84-85 originated from the same premises

[k]Carcasses 105-106 originated from the same premises

[l]Carcasses 109-110 originated from the same premises
